# Validation of the 1,4‐butanediol thermoplastic polyurethane as a novel material for 3D bioprinting applications

**DOI:** 10.1002/btm2.10192

**Published:** 2020-11-14

**Authors:** Carlos Chocarro‐Wrona, Juan de Vicente, Cristina Antich, Gema Jiménez, Daniel Martínez‐Moreno, Esmeralda Carrillo, Elvira Montañez, Patricia Gálvez‐Martín, Macarena Perán, Elena López‐Ruiz, Juan Antonio Marchal

**Affiliations:** ^1^ Biosanitary Research Institute of Granada (ibs.GRANADA) University Hospitals of Granada‐University of Granada Granada Spain; ^2^ Biopathology and Regenerative Medicine Institute (IBIMER), Centre for Biomedical Research (CIBM), University of Granada Granada Spain; ^3^ Department of Human Anatomy and Embryology Faculty of Medicine, University of Granada Granada Spain; ^4^ Excellence Research Unit “Modeling Nature” (MNat) University of Granada Granada Spain; ^5^ Department of Applied Physics Faculty of Sciences, University of Granada Granada Spain; ^6^ Biomedical Research Institute of Málaga (IBIMA) Málaga; ^7^ Department of Orthopedic Surgery and Traumatology Virgen de la Victoria University Hospital Málaga Spain; ^8^ Department of Pharmacy and Pharmaceutical Technology School of Pharmacy, University of Granada Granada Spain; ^9^ Advanced Therapies Area Bioibérica S.A.U Barcelona Spain; ^10^ Department of Health Sciences University of Jaén Jaén Spain

**Keywords:** 1,4‐butanediol thermoplastic polyurethane, 3D bioprinting, elastomer, MSCs, tissue engineering

## Abstract

Tissue engineering (TE) seeks to fabricate implants that mimic the mechanical strength, structure, and composition of native tissues. Cartilage TE requires the development of functional personalized implants with cartilage‐like mechanical properties capable of sustaining high load‐bearing environments to integrate into the surrounding tissue of the cartilage defect. In this study, we evaluated the novel 1,4‐butanediol thermoplastic polyurethane elastomer (b‐TPUe) derivative filament as a 3D bioprinting material with application in cartilage TE. The mechanical behavior of b‐TPUe in terms of friction and elasticity were examined and compared with human articular cartilage, PCL, and PLA. Moreover, infrapatellar fat pad‐derived human mesenchymal stem cells (MSCs) were bioprinted together with scaffolds. in vitro cytotoxicity, proliferative potential, cell viability, and chondrogenic differentiation were analyzed by Alamar blue assay, SEM, confocal microscopy, and RT‐qPCR. Moreover, in vivo biocompatibility and host integration were analyzed. b‐TPUe demonstrated a much closer compression and shear behavior to native cartilage than PCL and PLA, as well as closer tribological properties to cartilage. Moreover, b‐TPUe bioprinted scaffolds were able to maintain proper proliferative potential, cell viability, and supported MSCs chondrogenesis. Finally, *in vivo* studies revealed no toxic effects 21 days after scaffolds implantation, extracellular matrix deposition and integration within the surrounding tissue. This is the first study that validates the biocompatibility of b‐TPUe for 3D bioprinting. Our findings indicate that this biomaterial can be exploited for the automated biofabrication of artificial tissues with tailorable mechanical properties including the great potential for cartilage TE applications.

**Abbreviations**
b‐TPUe: 1,4‐butanediol thermoplastic polyurethane
CAD: computer‐aided design
CAM: computer‐aided manufacturing
DMMB: dimethylmethylene blue
ECM: extracellular matrix
ESEM: environmental scanning electron microscope
FBS: fetal bovine serum
GAGs: glycosaminoglycans
ITS: insulin‐transferrin‐selenium
MSCs: mesenchymal stem cells
OA: osteoarthritis
P/S: penicillin/streptomycin
SF: synovial fluid
OPD: ortho‐phenyldiamine
PBS: phosphate‐buffered saline
PCL: poly‐ɛ‐caprolactone
PLA: poly‐l‐lactic acid
PLGA: polylactic‐co‐glycolic acid
SEM: scanning electron microscope
SLA: stereolithography
TE: tissue engineering
TGF‐β3: transforming growth factor β3

## INTRODUCTION

In the last few years the 3D bioprinting technology has shown promising results in the biofabrication of artificial tissues for tissue engineering (TE) applications.^1^ This emerging technology uses computer‐aided design (CAD) and computer‐aided manufacturing (CAM) techniques, which in combination with the layer‐by‐layer fabrication nature of 3D printing, allows to create structures with different geometries while controlling the spatial distribution of cells, biomaterials, and growth factors.^2^, ^3^ Furthermore, 3D bioprinting brings advantages to the clinical field such as shorter fabrication time, higher precision than conventional TE techniques, and tailored production.^4^

Among 3D bioprinting techniques, extrusion‐based is the most extended as it offers the possibility to print a wide variety of biomaterial viscosities and is the most adaptable technology to be transferred to the clinical field.^5^, ^6^ Additionally, there are several commercially available extrusion‐based bioprinters, and they can also be adapted for testing novel biomaterials. Although this approach holds great promises for TE and regenerative medicine, as an emerging technology it also entails some bottlenecks. One of the main challenges is the restricted accessibility of materials necessary to produce constructs that can properly mimic the native tissue properties. The most common type of material used for this purpose are hydrogels, since they can offer a suitable 3D microenvironment that mimics the extracellular matrix (ECM) of natural tissues, promoting cell attachment and proliferation.^7^ However, hydrogel scaffolds usually lack mechanical strength and structural integrity, therefore, their mechanical properties need to be tuned or combined with synthetic stiffer biomaterials to enhance its mechanical properties.^8^

Several synthetic materials such as PLA,^9^, ^10^, ^11^ PCL^12^, ^13^, ^14^, ^15^ or polylactic‐co‐glycolic acid (PLGA)^16^, ^17^, ^18^, ^19^ have been used to generate bioprinted scaffolds for TE applications. However, these materials do not easily achieve to mimic the native tissue mechanical characteristics. The stiffness of porous scaffolds produced using rigid biomaterials, such as PLA,^20^ are in the MPa magnitude order comparable to those found in hard tissues such as porous bone.^21^ Therefore, significant efforts are being made for engineering flexible tissues that suffer mechanical loading such as ligaments, tendons, cartilage, blood vessels, skin, or muscles.^22^

In this sense, cartilage, as an avascular and stratified tissue, presents a limited capacity of repair, therefore, a severe damage will often require surgical intervention. However, the clinical surgical treatments such as ACI or MACI, which use a bilayer type I/III collagen membrane, lack long‐term effectiveness.^23^, ^24^, ^25^ Mosaicplasty, a treatment for focal chondral lesions, shows results that are relatively acceptable for the first 2 years but develops a sudden failure rate (approximately 55%) over the successive 2 years.^26^ Currently, the strategies for cartilage repair are concentrated on the creation of a complex material that biologically mimics the native tissue and get close to its biomechanical properties. Hence, many biomaterials, such as fibrin, silk, hyaluronic acid, chitosan, PLA, or PCL^27^ are being used to create scaffolds for cartilage TE, but, on one hand, natural‐based materials do not show enough integrity, and on the other hand, synthetic‐based materials do not have similar mechanical properties to cartilage such as friction and elasticity which limits their effectiveness and integration in the injury.^28^, ^29^

Polyurethane elastomers are a type of adaptable synthetic materials broadly applied to biomedical purposes because of their biocompatibility and good mechanical properties.^30^, ^31^, ^32^ Recently, a novel elastic 3D printing filament consistent of a 1,4‐butanediol thermoplastic polyurethane (b‐TPUe) derivative shows a combination of mechanical properties that makes it a promising candidate for TE.^33^

In this study, we evaluate, for the first time, the potential use of b‐TPUe filament as a new 3D bioprinting material for biomedical applications. We carried out a rheological characterization to analyze their mechanical properties (in shear and compression) and a tribological study to evaluate the frictional behavior in synovial fluid‐lubricated b‐TPUe‐cartilage tribopairs. Moreover, we compared *in vitro* and *in vivo* the biocompatibility of b‐TPUe 3D printed scaffolds versus PCL and showed the potential application of this material for cartilage TE. Finally, we described the induced chondrogenic differentiation of MSCs isolated from infrapatellar fat pad when cultured in 3D bioprinted b‐TPUe scaffolds. In conclusion we present a novel use of b‐TPUe filament with potential to support the development of cartilage‐like phenotype as a promising TE biomaterial.

## RESULTS

### Fabrication of scaffolds

**FIGURE 1:**
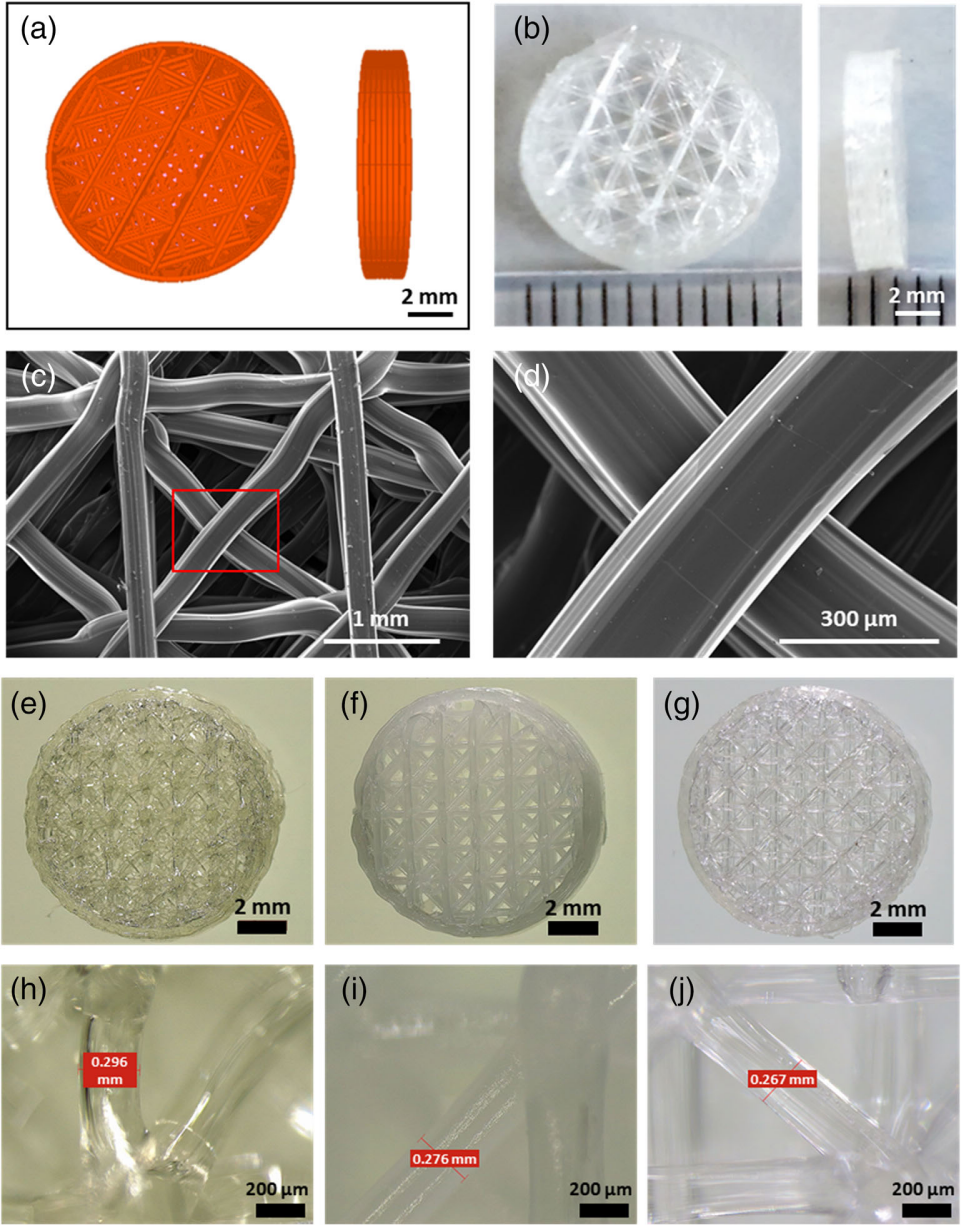
B‐TPUe scaffolds design: (a) CAD model of the scaffold design. (b) 3D printed b‐TPUe scaffold (10 mm in diameter and 3 mm in height). (c and d) SEM images of the top surface of b‐TPUe 3D printed scaffolds (scale bars: 1 mm and 300 μm, respectively). (e–g) Macroscopic view of b‐TPUe, PCL and PLA scaffolds, respectively. (h–j) Scaffold fiber width of b‐TPUe, PCL, and PLA scaffolds, respectively

3D b‐TPUe scaffolds were designed with a regular geometry and structure to enable an adequate cell bioprinting **(**Figure 1a **)** and successfully fabricated with the desired shape and dimensions, like the CAD model **(**Figure 1b **)**. Scanning electron microscope (SEM) images **(**Figure 1c,d **)** show the obtained scaffold pores and filament surfaces and demonstrate that the thickness of the fibers of the b‐TPUe printed scaffolds (200–400 μm) is maintained during the fabrication process **(**Figure 1e–j **)**. As can be clearly seen, the pores are large, ranging from 500 to 700 μm **(**Figure 1c,d **)** and have a regular structure, uniformly distributed, and interconnected.

### Frictional test

**FIGURE 2:**
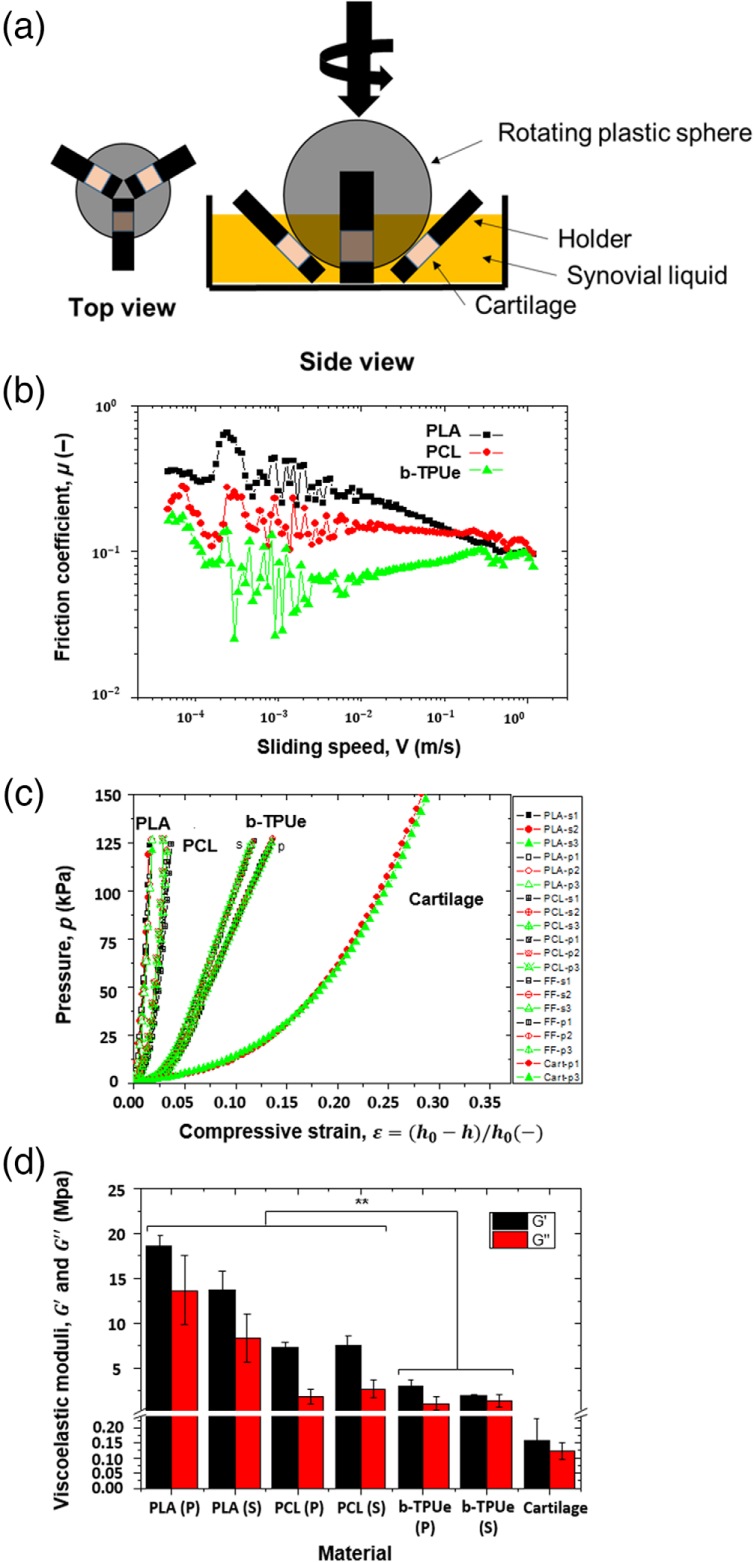
Tribological and rheological characterization. (a) Schematic diagram of the tribological set‐up. (b) Frictional behavior of PLA (black), PCL (red), and b‐TPUe (green). (c) Compression curves corresponding to the studied samples (s: solid; p: porous). (d) Linear viscoelastic moduli (*G*′ and *G*″) for the materials studied (**p < 0.01). Graphs created using the Origin 9.0 software

The frictional behavior of the different plastics used in this work is exemplified in Figure 2a,b. For this, plastic‐cartilage point contacts were lubricated by synovial fluid and data are plotted in terms of a Stribeck curve, where friction coefficient is represented as a function of the sliding speed for a constant normal load of 1 N. Only for b‐TPUe, the contact operates in the full film lubricated regime as demonstrated by the increase in friction for large sliding speeds. As seen in Figure 2b, a lower friction was measured for b‐TPUe, with average friction coefficients (*μ*) under 0.1, followed by PCL and PLA, with average *μ* above 0.1.

### Compression test

The mechanical behavior of b‐TPUe was examined and compared with human articular cartilage, PCL, and PLA. The compression curves of PLA, PCL, b‐TPUe and cartilage are shown in Figure 2c. These strongly nonlinear curves clearly demonstrate that b‐TPUe is more compliant than the other materials investigated (PLA and PCL). Also, unlike PLA and PCL, results for solid (s) and porous (p) b‐TPUe scaffolds showed different behaviors in compression. Interestingly, porous b‐TPUe scaffolds were significantly softer than their solid counterparts, suggesting that b‐TPUe scaffold elasticity can be tailored by changing the porosity. So, b‐TPUe scaffolds with greater porosity present a mechanical behavior closer to the one of native cartilage. In addition, for low strains ε, the mechanical behavior of b‐TPUe was closer to that observed in natural cartilage when compared with PCL or PLA. Moreover, the shear moduli obtained in the second interval of the test showed a clear correlation with the compression data, again demonstrating that b‐TPUe exhibited a much lower storage modulus in contrast to the conventional plastics, PCL, and PLA **(**Figure 2d **)**.

### Effects of b‐TPUe‐conditioned medium on MSCs proliferation

**FIGURE 3:**
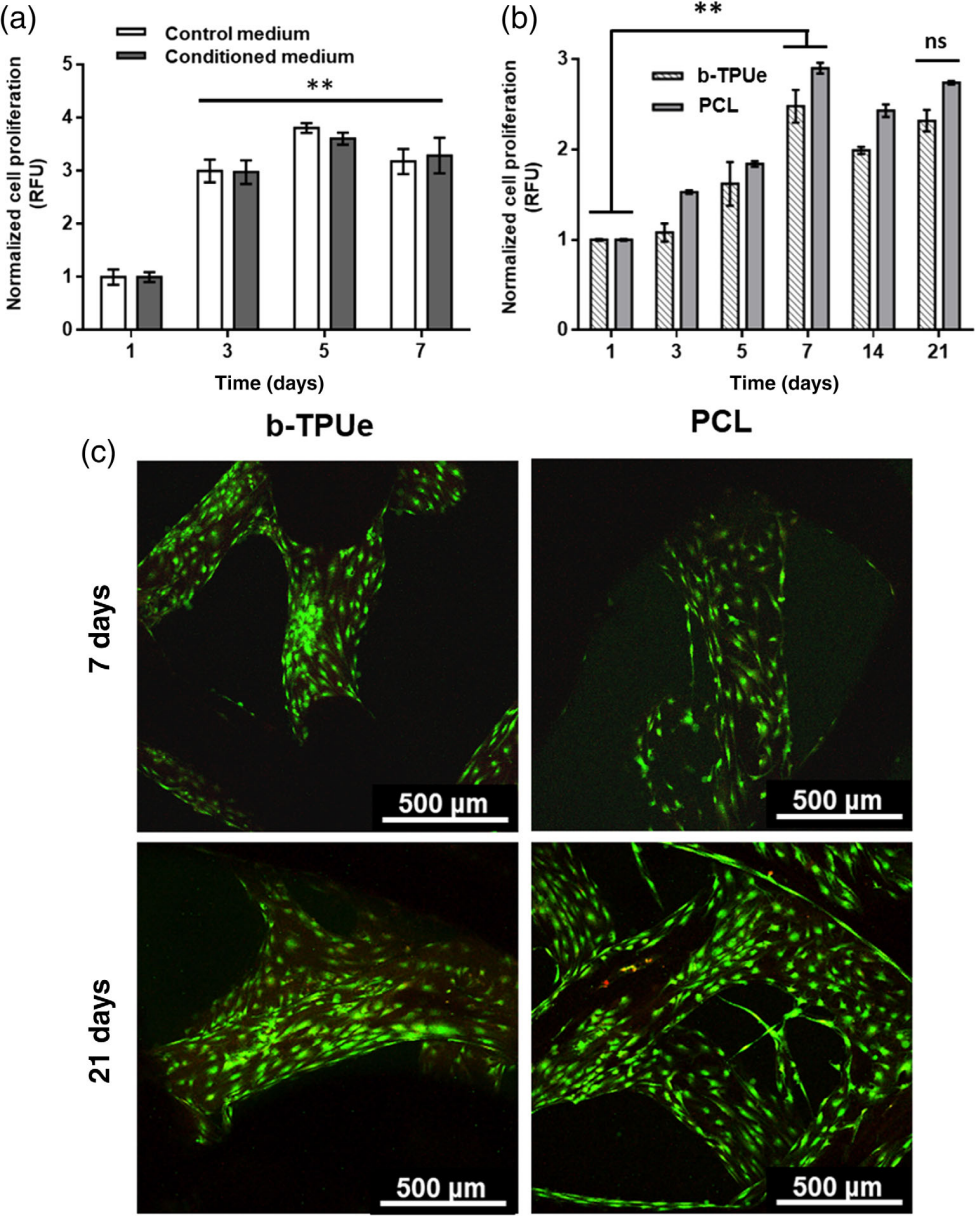
*In vitro* biocompatibility of b‐TPUe bioprinted scaffolds with MSCs. (a) Proliferative potential of MSCs cultured with control (DMEM 10% FBS, 1% P/S) or b‐TPUe‐conditioned medium up to 7 days (**p < 0.01). (b) MSCs proliferation cultured in both b‐TPUe and PCL bioprinted scaffolds up to 21 days with no significant differences between PCL and b‐TPUe (no significance: ns). Significant cell growth was observed in both materials at day 7 of culture in both materials (**p < 0.01) (RFU: relative fluorescence units). (c) Representative confocal images of MSCs grown in both b‐TPUe and PCL bioprinted scaffolds at day 7 and 21. Live/dead assay was employed, using calcein (green) and ethidium homodimer (red), live cells were stained green while dead cells were stained red. Scale bars: 500 μm. Graphs created using the GraphPad Prism 6.01 software

We conducted a proliferation assay to evaluate if the exposure to b‐TPUe could have a negative effect in the proliferative potential of MSCs. Results showed no adverse effects in the proliferative potential of MSCs cultured in b‐TPUe‐conditioned medium for 7 days when compared with MSCs cultured with control medium **(**Figure 3a **)**.

### Proliferation and viability of MSCs cultured in b‐TPUe bioprinted scaffolds

Cell proliferation of MSCs cultured in b‐TPUe bioprinted scaffolds was evaluated with an AlamarBlue® assay. PCL filament was used as a control material since it is a reference biomaterial used in cartilage bioprinting.^34^, ^35^, ^36^, ^37^ As can be observed in Figure 3b, cell proliferation increased from day 1 to day 21 with a significant increase at day 7 of culture in both bioprinting materials, while at day 21 no significant differences were observed in the proliferation rate between cells printed in b‐TPUe and those in PCL control scaffolds **(**Figure 3b **)**.

The viability of MSCs was also evaluated to validate the biocompatibility of b‐TPUe printed scaffolds using a live/dead assay. Confocal images **(**Figure 3c **)** show a majority of green viable MSCs covering both b‐TPUe and PCL scaffold fiber surfaces at day 7 and 21 after bioprinting that indicates that b‐TPUe bioprinted scaffolds can supports MSCs growth in a same manner as PCL.

### Chondrogenic differentiation of MSCs cultured in b‐TPUe bioprinted scaffolds

**FIGURE 4:**
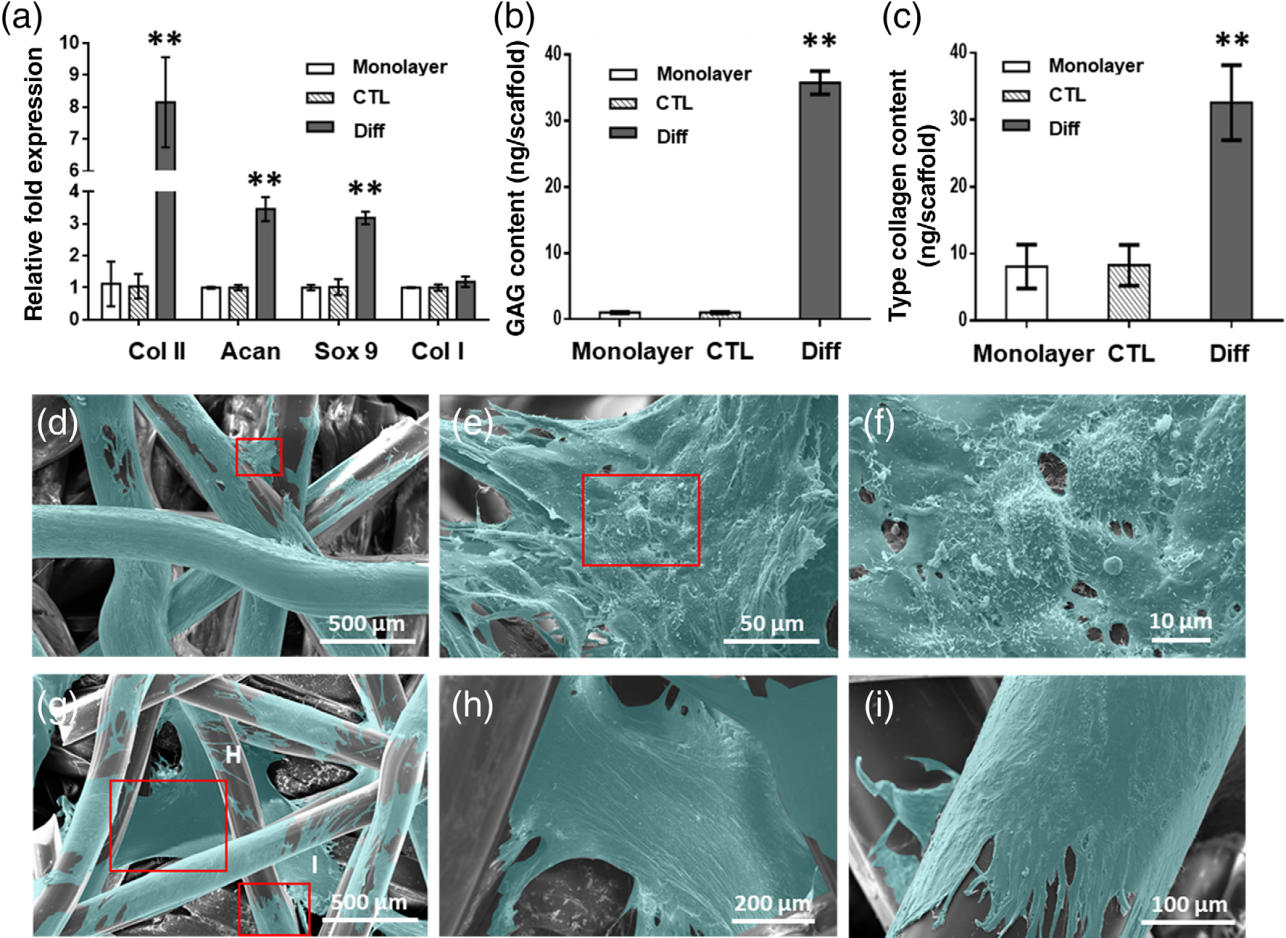
MSCs chondrogenic differentiation in b‐TPUe bioprinted scaffolds. Chondrogenic differentiation was evaluated in MSCs cultured in monolayer, b‐TPUe scaffolds (CTL), and b‐TPUe scaffolds under differentiation conditions (Diff) after 21 days in culture. (a) RT‐PCR analysis of chondrogenic key markers. (b) GAGs quantification. (c) Type II collagen quantification. (d–f) SEM representative images of MSCs growing in b‐TPUe bioprinted scaffolds at day 21 (**p < 0.01). (g–i) SEM representative images of MSCs growing in b‐TPUe bioprinted scaffolds under chondrogenic differentiation conditions. Scale bars: 500 μm (d), 50 μm (e), 10 μm (f), 500 μm (g), 200 μm (h), 100 μm (i). Graphs created using the GraphPad Prism 6.01 software. SEM images false‐colored using the cross‐platform image editor GIMP (version 2.10.14)

The MSCs employed in this study were isolated from human adipose tissue. Differentiation potential and phenotypic characterization of isolated MSCs are shown in Supporting Information Figure 1. To investigate the capacity of b‐TPUe scaffolds to support the induction of cartilage‐like phenotype, chondrogenic key markers were evaluated by RT‐PCR after 21 days of culture of bioprinted cell‐seeded b‐TPUe scaffolds under chondrogenic conditions. Cells extracted from b‐TPUe bioprinted scaffolds cultured under chondrogenic media showed a significant increment in type II collagen, aggrecan, and Sox9 gene expression when compared with cells grown in monolayer and onto b‐TPUe scaffolds without chondrogenic media **(**Figure 4a **)**.

The ECM produced under induction of chondrogenic differentiation was evaluated assessing glycosaminoglycans (GAGs) and type II collagen concentration in cell culture supernatants of MSCs monolayers and printed MSCs b‐TPUe scaffolds cultured with (Diff) or without (CTL) chondrogenic medium for 21 days. The GAGs analysis showed that b‐TPUe printed scaffolds in chondrogenic conditions produced a high significant number of GAGs compared to control b‐TPUe scaffolds or monolayer conditions **(**Figure 4b **)**. Similarly, collagen type II production was also markedly greater in b‐TPUe printed scaffolds cultured under chondrogenic conditions at 21 days compared to control b‐TPUe scaffolds and monolayer conditions **(**Figure 4c **)**.

Moreover, SEM images showed cell growth and wide cell spread throughout the scaffold over the b‐TPUe filament after 21 days of cell growth with and without differentiation conditions. It is relevant to note that cells attached to the filament surface and junctions via formation of filopodia and started to form a network of cell and matrix **(**Figure 4d–f **)**. Also, an enhanced cell growth that covered the pore spaces **(**Figure 4g,h **)** and over the filament surfaces was observed **(**Figure 4i **)**.

### *In vivo* assay

**FIGURE 5:**
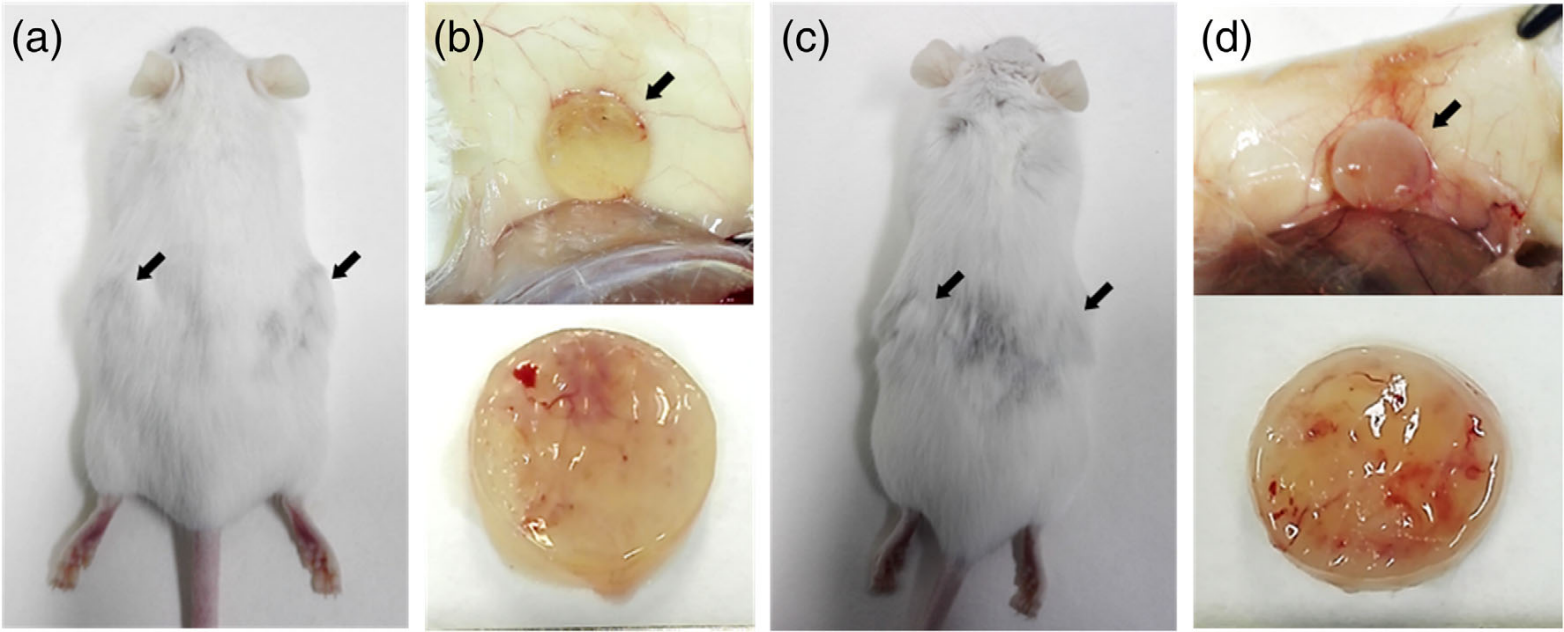
*In vivo* biocompatibility of b‐TPUe. (a) Macroscopic image of the locations of implanted b‐TPUe scaffolds in CD1 mice. Scaffolds were implanted in the dorsal region of 8 weeks old CD1 mice and resected 21 days after surgery procedure. (b) Images of b‐TPUe scaffolds recovered from CD1 mice. (c) Macroscopic image of the locations of implanted PCL scaffolds in CD1 mice. (d) Images of PCL scaffolds implanted in the dorsal region of CD1 mice

Biocompatibility of cell‐free b‐TPUe scaffolds was assessed *in vivo* by subcutaneous *in situ* implantation in the back of immunocompetent CD‐1 mice using PCL as control material **(**Figure 5a–d **)**. During the study, no cases of mice showing pain behavior that could be induced by the scaffold implantation or infection were observed. The scaffolds were excised 21 days after implantation, and both scaffolds and mice were photographed to evaluate their appearance and integration within the subcutaneous surrounding tissue. Both b‐TPUe and PCL scaffolds were firmly anchored and integrated within the subcutaneous tissue maintaining their shape and integrity. Moreover, no sign of edema or macroscopic inflammation was detected **(**Figure 5b,d **)**.

**FIGURE 6:**
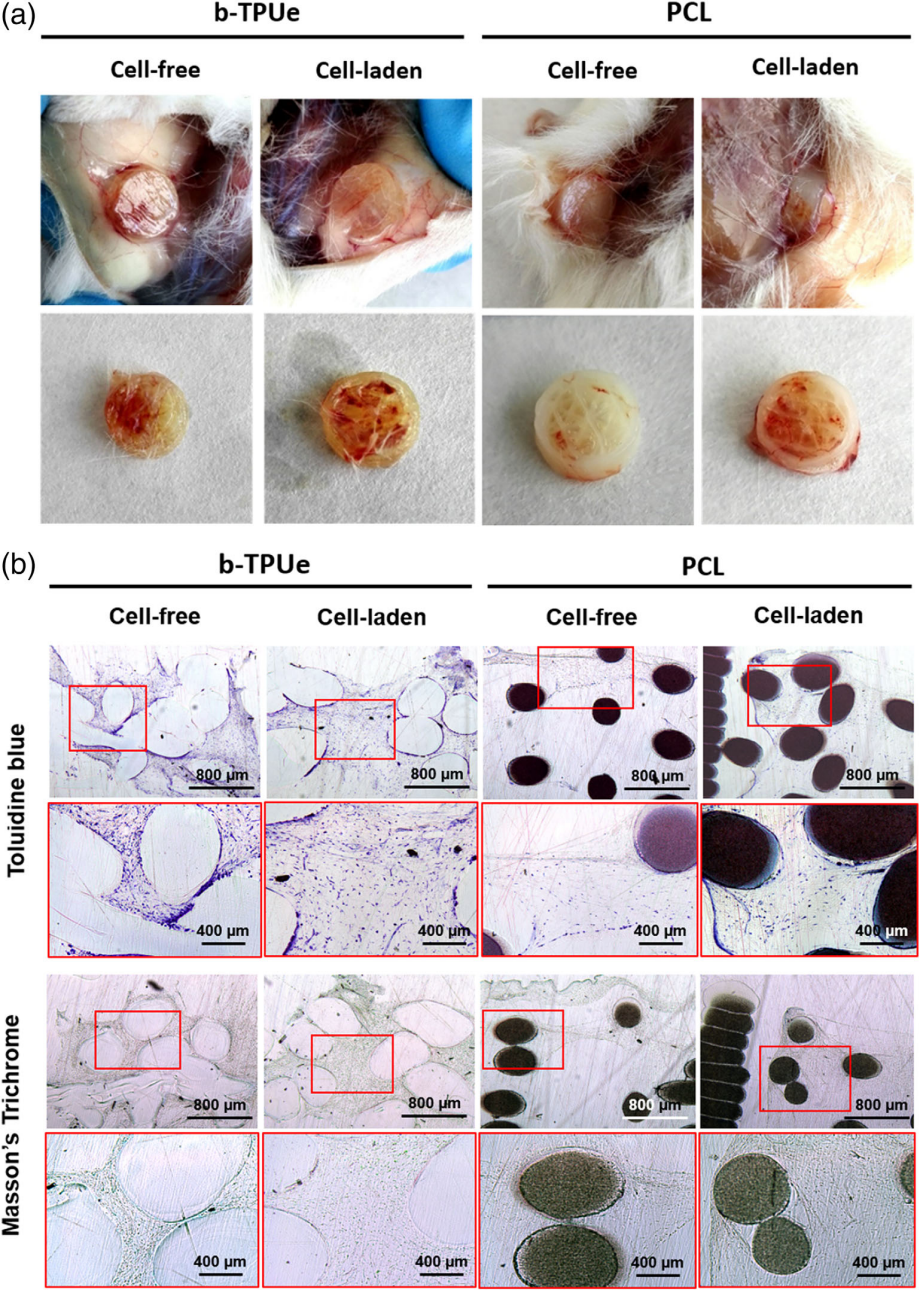
*In vivo* biocompatibility of b‐TPUe bioprinted scaffolds with MSCs. (a) Macroscopic images for cell‐free and cell‐laden b‐TPUe and PCL scaffolds fabricated by 3D bioprinting. Scaffolds were implanted in the dorsal region of 8 weeks old female NSG mice and resected 21 days after surgery procedure. (b) Histologic analysis of Toluidine blue and Masson's Trichrome staining of cell‐free and cell‐laden b‐TPUe and PCL scaffolds 3 weeks postimplantation. Scale bars: 800 μm for black‐labeled images, and 400 μm for red‐labeled images

To assess the integration of the scaffolds within the surrounding tissue, both b‐TPUe‐MSCs and PCL‐ MSCs bioprinted scaffolds cultured for 21 days were transplanted into subcutaneous tissue on the flanks of immunodeficient NSG mice, as well as b‐TPUe and PCL cell‐free scaffolds, and harvested 3 weeks later for subsequent analysis. The implanted bioprinted cell‐laden scaffolds showed good integration at the surrounding tissue 21 days postimplantation **(**Figure 6a **)**. Moreover, cell‐free scaffolds showed that host cells infiltrated, and grew into the scaffold. Toluidine blue staining demonstrated the presence of GAGs in both b‐TPUe and PCL scaffolds. Masson's Trichrome staining showed that the deposition of collagenous fibers occurred in both materials and in both cell‐free and cell‐laden conditions **(**Figure 6b **)**.

## DISCUSSION

The 3D bioprinting technology allows high precision, fabrication, and customized production, which are important features for biomedical applications. Traditional methods for scaffold manufacturing comprise phase separation,^38^ electrospinning,^39^ freeze‐drying,^40^ and gas forming.^41^ Comparing this methods to 3D bioprinting, they lack a high precision control of the pore size and shape.^42^

In this study, a polyurethane‐based 3D printing material, b‐TPUe, was successfully used to fabricate scaffolds by 3D bioprinting that were able to maintain cellular viability and growth. We selected the b‐TPUe since it belongs to the polyurethane thermoplastics, an adaptable category of materials broadly used for biomedical purposes thanks to their biocompatibility, elasticity and strength.^43^, ^44^, ^45^, ^46^, ^47^ There are other materials which are designed to fill and integrate irregular cartilage wounds, and are also already being tested in clinical trials,^48^ such as PEG‐based adhesive‐hydrogels composites; however, they do not show similar mechanical properties to native cartilage, because they previously have to maturate and produce ECM with the same properties of the surrounding healthy tissue. Moreover, to be immobilized on the tissue surface of the lesion they need of a light‐initiated reaction to cross‐link the liquid polymer solution. In contrast, our b‐TPUe polymer can be used to bioprint personalized scaffolds adapted to the shape of the patient injury. In fact, polyurethane‐based materials are already being tested in clinical trials.^49^, ^50^ 3D bioprinting technology allows us to fabricate b‐TPUe scaffolds with the desired thickness of fiber and pore size, biomimicking the tissue microstructure, and thus ameliorating the integration of the scaffold within the specific location. The porosity and interconnectivity of the scaffold plays a significant role in nutrient supply, gas diffusion and metabolic waste removal.^51^, ^52^ Therefore, cells can penetrate the pores following their growth on the scaffold.^53^

A selected biomaterial for treating joint replacements is expected to preserve the remaining native cartilage from degradation while maintaining the frictional properties of the joint.^54^ Analyzing the friction profile of the studied materials, b‐TPUe showed to exert less friction toward the native cartilage surface than PLA and PCL, showing *μ* values closer to the cartilage‐to‐cartilage interaction.^55^ Also, the mechanical properties of a scaffold are important for engineering tissues, especially for cartilage, which is subjected to cyclic mechanical forces.^56^ Although scaffolds based on hydrogels mimic more adequately the mechanical properties found in native tissues,^57^ their compressive modulus are typically an order of magnitude less than native cartilage tissue.^58^, ^59^ Otherwise, scaffolds produced with thermoplastics possess higher Young's modulus than those based on hydrogels.^60^, ^61^ The obtained results suggests that b‐TPUe scaffold elasticity can be tailored, by changing the porosity, to achieve closer values to the natural cartilage Young's modulus than hydrogel scaffolds and synthetic polymers such as PCL or PLA,^57^ thus exhibiting promising customizable mechanical properties. The viscoelastic modulus in scaffold‐based TE is important to approximate and supply the unique properties of the normal articular cartilage that is trying to be replaced.^42^ The ideal scaffold for cartilage regeneration is a material with viscoelastic and hydrodynamic properties that mimic the mechanical microenvironment of cartilage matrix, which could provide proper mechanical and biochemical signals for chondrocyte adhesion proliferation, differentiation, and ECM formation.^62^ Moreover, this similarity to the natural viscoelastic properties and compliance with dynamic environments is important for the integration without damaging the surrounding tissue. In fact, recent researches noticed the importance of material viscoelasticity in cartilage TE,^63^, ^64^ since viscoelastic matrix with stress relaxation could mimic the mechanical microenvironment of soft tissues, and thus favor chondrogenic differentiation and a better integration with the cartilage.^65^

Biocompatibility must be a priority when selecting biomaterials for TE.^66^ Polyurethanes are considered to have good biocompatibility properties and are widely used for long‐term medical implants, such as cardiac pacemakers and vascular grafts.^67^ Since b‐TPUe is a recently developed polyurethane‐based 3D printing filament, no previous data concerning the possible cytotoxicity of this material on cell growth has been previously published. Results of the cytotoxicity, proliferation and viability assays showed no cytotoxic effects of b‐TPUe, suggesting that it can provide an environment that supports MSCs proliferation in a same manner as PCL.^68^ In fact, large spaces between the fibers allowed the adhered cells to start accommodating between the stacking fibers.

Regarding cartilage ECM production, expression of type II collagen and aggrecan, which are the main proteins of the hyaline cartilage ECM,^69^ showed to be upregulated in cells cultured in b‐TPUe scaffold under chondrogenic media compared with control conditions. Similarly, Sox9, which is a known transcription factor of chondrogenesis that acts in the early stages of chondrogenic differentiation inducing type II collagen production^70^ also showed to be upregulated. In addition, non‐increased expression of collagen type I in b‐TPUe scaffolds under chondrogenic media compared with their counterparts cultured in non‐differentiated media or cells cultured in monolayer without chondrogenic media was observed. Type I collagen has been described in fibroblastic differentiation and could indicate the formation of fibrous cartilage.^71^ The upregulation of chondrogenic genes, together with the low expression of collagen type I of MSCs bioprinted in b‐TPUe scaffolds indicate the ability of this material to support the differentiation of MSCs into chondrocyte‐like cells. In accordance with these results, an increased GAGs and collagen type II deposition in the ECM of b‐TPUe MSCs bioprinted scaffolds cultured under chondrogenic conditions indicated the development of a cartilaginous‐like matrix.^72^ This chondrogenic support of b‐TPUe MSCs bioprinted scaffolds are in agreement with those previously obtained for PCL.^73^ Further studies are necessary to compare the MSCs chondrogenesis of b‐TPUe with other biomaterials such as PCL, PLA or PEG‐based hydrogels, among others.

In the present study, we tried to evaluate qualitatively the macroscopic response to b‐TPUe scaffolds in an *in vivo* environment. The lack of pain behavior, infection, edema, or macroscopic tissue inflammation during the *in vivo* assay with immunocompetent CD‐1 mice, as well as the maintenance of shape and integrity of the scaffold, and its integration within the implantation surrounding tissue indicate the *in vivo* biocompatibility of b‐TPUe as previously described for other 3D polyurethanes.^74^ Similarly, when implanted in immunodeficient NSG mice, the deposition of collagenous fibers in both cell‐free and cell‐laden scaffolds suggest that b‐TPUe can allow *in vivo* GAGs and collagenous fiber production as well as PCL. Thus, it can be stated that b‐TPUe polymer scaffolds showed good *in vivo* ECM deposition confirming the integration of b‐TPUe within the host's tissue.^75^

## CONCLUSION

In this study, a novel elastic polyurethane‐based 3D printing material, b‐TPUe, was successfully used to fabricate 3D printed scaffolds with improved rheological and tribological properties compared to PCL and PLA. This new printing material, besides showing the ability to support the growth and chondrogenic differentiation of MSCs, also presents a mechanical behavior closer to natural cartilage when compared with PCL and PLA. Interestingly, the elastic characteristics of b‐TPUe changes when modifying the porosity, improving the customization of the mechanical properties of the constructs, therefore offering the possibility to better adapt this parameter to the desired target tissue. Moreover, b‐TPUe showed a tribological performance closer to cartilage in comparison to PLA and PCL, suggesting that it is an appropriate material to be used in cartilage replacement to restore joint function. Furthermore, b‐TPUe demonstrated a high biocompatibility when growing MSCs onto b‐TPUe scaffolds. In fact, b‐TPUe bioprinted scaffolds were found to support MSCs proliferation and the upregulation of hyaline‐like cartilage tissue markers in their gene expression, with no *in vivo* toxic effects. These results highlight the potential of b‐TPUe as a 3D printing material with application in cartilage TE. In addition, the development of materials such as b‐TPUe that allow the scaffold customization is essential for other soft tissues such as tendon, muscle, or ligaments. Moreover, due to the excellent biomechanical properties and biocompatibility, we have set the basis for further exploration of this novel material for biomedical and tissue regenerative applications.

## EXPERIMENTAL SECTION

### Patients

Human infrapatellar fat pad, cartilage tissue and synovial fluid were obtained from patients with knee OA during joint replacement surgery. Ethical approval for the study was obtained from the Ethics Committee of the Clinical University Hospital of Málaga, Spain (ethic permission number: 02/022010 Hospital Virgen de la Victoria, Málaga). Informed patient consent was obtained for all samples used in this study. None of the patients had a history of inflammatory arthritis or crystal‐induced arthritis. Hoffa's fat pad was harvested from the inside of the capsule excluding vascular areas and synovial regions. Human articular cartilage was obtained from the femoral side, selecting the non‐overload compartment. Only cartilage that looked normal macroscopically was used for this study. Samples collected at joint arthroplasty were transported to the laboratory in Dulbecco's modified Eagle's medium (DMEM; Sigma, St. Louis, MO, USA) with 1% penicillin/streptomycin (P/S). Synovial fluid (SF) was pooled from knee joints and mixed on an orbital shaker. Only samples that were free of blood contamination were used, as assessed visually. SF was stored at −20°C between testing sessions.^76^

### Isolation and culture of human MSCs from infrapatellar fat pad

Infrapatellar fat pad tissue was minced and digested with an enzymatic solution of 1 mg/ml collagenase type IA (Sigma) and incubated in shaking at 37°C for 2 h. Once digested, collagenase was removed with a single wash of sterilized phosphate‐buffered saline (PBS), followed by two washes of DMEM supplemented with 10% fetal bovine serum (FBS) (Sigma). The cell pellet was resuspended in DMEM supplemented with 10% FBS and 1% P/S, placed into tissue culture flasks, and cultured at 37°C in a 5% CO_2_ atmosphere. After 48 h the medium was removed to discard non‐adherent and dead cells.^77^ When 80% of confluence was reached, cells were released with Tryple Express (Gibco) and subcultured. Phenotype and differentiation potential of isolated MSCs were characterized, as previously described.^77^, ^78^ To examine their immunophenotype, MSCs were harvested, rinsed twice in PBS, and blocked with bovine serum albumin (BSA) 1% in PBS for 10 min at room temperature (RT). Fluorescent monoclonal antibodies CD73, CD90 and CD105 antibodies were used as positive markers, while CD34, CD45 and HLA‐DR antibodies were used as negative markers.^79^ Antibodies were incubated at dark for 15 min at 4°C. Before their analysis, MSCs were washed twice with PBS to remove non‐bound antibodies. Flow cytometry analysis was performed with a BD FACSCantoTM II cytometer (BD Biosciences, CA, USA) and the resulting data were analyzed using the FACSDivaTM software (BD Biosciences). For the differentiation assays, MSCs were plated at 1 × 10^5^ cells/cm^2^ in DMEM supplemented with 10% FBS and 1% P/S into 6‐well culture plates. After 48 h, the culture medium was replaced with specific differentiation‐inductive medium. For adipogenic, osteogenic, and chondrogenic differentiation, MSCs were cultured for 2 weeks in MSC Adipogenic Differentiation BulletKit™ (Lonza, Basilea, Switzerland), MSC Osteogenic Differentiation BulletKit™ (Lonza), and StemMACS ChondroDiff Medium (Miltenyi Biotec, Bergisch Gladbach, Germany), respectively. Differentiated cell cultures were stained with oil red O (Amresco, Solon, OH, USA) for adipogenic differentiation, alizarin red (Lonza) for osteogenic differentiation or toluidine blue (Sigma‐Aldrich) for chondrogenic differentiation.

### Bioprinting process

A Regemat 3D V1 bioprinter (REGEMAT 3D S.L., Spain) was used for 3D printing with a direct extruder to fabricate the scaffolds.^80^ Commercial PCL (3D4Makers, 1.75 mm filament, printing temperature: 70–90°C; semicrystalline aliphatic polyester) was melted at 75°C and printed at rate of 1.1 mm/s. Commercial PLA (Smart Materials 3D, Spain, 1.75 mm filament, printing temperature: 190–210°C; polymerized polylactic acid) was melted at 200°C and printed at rate of 1.2 mm/s. Commercial b‐TPUe (Recreus Industries s.l., 1.75 mm filament, printing temperature: 200–230°C; based on methylene diphenyl diisocyanate [MDI] and 1,4‐butanediol) was melted at 200°C and printed at rate of 1.4 mm/s. Printing parameters were optimized for each material in order to obtain the best printability and scaffold quality layout. PCL, PLA, and b‐TPUe scaffolds were designed to be extruded with triangular patterns for the infill with a pore size of 0.6 mm, solid walls consisting of a perimeter of 0.4 mm width, and three solid layers for the bottom, with a 0.2 mm layer height (Figure 1a). The scaffolds were printed as 3D cylindrical frameworks in a triangular inner lattice from alternately stacking filament fibers (Figure 1b). For 3D bioprinting with cells, the Regemat 3D V1 bioprinter was placed in a laminar flow hood. In the same process, the thermal extruder unit of the bioprinter was used to print the scaffolds, and then the syringe unit of the bioprinter was used to seed the cell suspension into the porous structure with a 200 μm diameter needle (1 × 10^5^ cells/scaffold). PCL was used as a control material instead of PLA as it is more used for biomedical purposes than its counterpart. For all *in vitro* assays the scaffold dimensions were designed to fit in a 24‐well plate (10 mm in diameter and 3 mm in height; 15 layers), with smaller dimensions for the *in vivo* assays (5 mm in diameter and 3 mm in height; 15 layers). Once bioprinted, the scaffolds were introduced in a 24‐well plate and incubated for at least 1 h to allow the cells to adhere to the fibers. Finally, the scaffolds were submerged in culture medium containing DMEM supplemented with 10% FBS and 1% P/S and then, stored at 37°C in a 5% CO_2_ atmosphere. Scaffolds used to support MSCs chondrogenic differentiation were cultured in DMEM supplemented with 10% FBS, 1% P/S, 50 μg/μl l‐ascorbic acid 2‐phosphate (Sigma), 40 μg/ml proline (Sigma), 1% insulin‐transferrin‐selenium (ITS) (Gibco), 40 μg/μl l‐proline (Sigma), and 10 ng/ml transforming growth factor β3 (TGF‐β3).^81^

### Tribological tests

A ball‐on‐three plates tribometer was adapted to a rheometer (Anton Paar, Austria) to interrogate the lubricating behavior of the different materials. The contact consisted in a plastic ball (made of PLA, PCL, or b‐TPUe) that slides along three cartilage surfaces (cartilage disks with a diameter of 5 mm) lubricated by synovial fluid. The MCR501 rheometer head (Anton Paar) was used to calculate the friction coefficient. A schematic diagram of the test set‐up is shown in Figure 2a. In this set‐up, a ball is pressed at a given normal force *F*_*N*_ against three plates that are mounted on a movable stage. The experimental protocol was as follows. First, the test rig was assembled, and 400 μl of SF was added. This amount was enough to fully immerse the three‐point contacts to a depth of 1 mm. Next, temperature was stabilized at 25°C and the plastic ball was loaded against the cartilage plates. Then, the ball was made to slide over the plates at a controlled (decreasing) speed *V*, from 2500 to 0.1 rpm under a normal force of *F*_*N*_ = 1 N (5 s per data point), while the resulting torque *T* sensed by the ball was monitored. The friction coefficient *μ* was computed with *μ* = *T*/(*F*_*N*_*R*) being *R* the radius of the ball.

### Rheological assays

Specimens for rheological assays were printed with 20 mm in diameter and 5 mm in height, solids and porous to analyze the effect of the infill over the mechanical characteristics of the scaffold (*n* = 3). Porous samples were printed with the same pattern of the ones used for cell culture tests. A MCR302 (Anton Paar) head was used to carry out rheological measurements at 25°C. A three‐step test was designed to obtain information on the compression and shearing characteristics of specimen. First, the scaffold was placed onto the base of the rheometer. Then, the rheometer head was approached at a constant speed (10 μm/s) up to a normal force of 40 N. Next, the specimen was oscillatory sheared according to a strain amplitude of 0.00001% at a frequency of 1 Hz and normal force of 40 N to determine the shear viscoelastic moduli and, finally, the upper plate was separated at a constant speed (10 μm/s).

### Cell viability assay

Cell viability in the 3D printed scaffolds was determined on days 7 and 21 after bioprinting using Live/Dead™ Viability/Cytotoxicity Kit (Invitrogen). The printed constructs were incubated in PBS containing calcein AM (2 μM) and ethidium homodimer (4 μM) at 37°C for 30 min to stain live and dead cells.^82^ Scaffolds were imaged by confocal microscopy (Nikon Eclipse Ti‐E A1, Amsterdam, Netherlands) and analyzed using NIS‐Elements software (Amsterdam, Netherlands).

### Scanning electron microscopy

The morphology and structure of b‐TPUe scaffolds were analyzed using a variable‐pressure and environmental scanning electron microscope (ESEM) FEI, mod. Quanta 400 (Oregon, USA). The analysis was performed in high vacuum mode to characterize the surface structure of scaffolds and cell growth. Samples were fixed with 2% glutaraldehyde and then, were rinsed in 0.1 M cacodylate buffer and incubated overnight at 4°C. For critical point, the samples were then maintained with Osmium tetroxide 1% RT during 1 h and dehydrated in a series of ethanol solutions (50%, 70%, 90%, 100%, 100%, 100%), by soaking the samples in each solution for 15 min. Subsequently, samples were critical point dried in a desiccator (Leica EMCPD300), and covered by evaporating them in a carbon evaporator (Emitech K975X).

### *In vitro* cytotoxicity test

MSCs culture medium aliquots were conditioned with b‐TPUe samples as previously described.^83^ Briefly, b‐TPUe sterilized scaffolds for a total mass of 3 g were placed in T‐75 tissue culture flasks and soaked in 100 mL of complete cell culture medium for 10 days at 37°C in a cell culture incubator on a rocking platform. Control medium was incubated in parallel, but without the b‐TPUe scaffolds. MSCs were plated in a 6‐well plate at 1 × 10^5^ cells/well. After 24 h the medium was replaced with a mix of a 1:1 fresh medium: b‐TPUe‐conditioned medium or with fresh control medium. Cell growth was analyzed at different time points: 1, 3, 5, and 7 days using AlamarBlue® assay (Bio‐Rad Laboratories, Inc., manufactured by Trek Diagnostic System). Cells were incubated with AlamarBlue® solution at 37°C for 3 h. Fluorescence of reduced AlamarBlue® was determined at 530/590 nm excitation/emission wavelengths (Synergy HT, BIO‐TEK).

### Cell proliferation assay

Cell proliferation was analyzed using AlamarBlue® assay after 1, 3, 5, 7, 14, and 21 days. The scaffolds were incubated with AlamarBlue® solution at 37°C for 3 h. Fluorescence of reduced AlamarBlue® was determined at 530/590 nm excitation/emission wavelengths.

### RNA isolation and real time‐PCR analysis

Total cellular RNA was isolated using TriReagent (Sigma) and reverse transcribed using the Reverse Transcription System kit (Promega). Real‐time PCR was performed using the SYBR‐Green PCR Master mix (Promega) according to the manufacturer's recommendations. PCR reactions were performed as follows: an initial denaturation at 95°C for 2 min, 40 cycles of 95°C for 5 s followed by 60°C for 30 s, and final cycle of dissociation of 60–95°C. The gene expression levels were normalized to corresponding GAPDH values and are shown as relative fold expression to the control sample. All samples were analyzed in triplicate for each gene. Primer sequences used are shown in Table 1.

**TABLE 1:** Primer sequences

Gene	Forward	Reverse
Col 1	ATGGATGAGGAAACTGGCAACT	GCCATCGACAAGAACAGTGTAAGT
Col 2	GAGACAGCATGACGCCGAG	GCGGATGCTCTCAATCTGGT
Acan	AGGATGGCTTCCACCAGTGC	TGCGTAAAAGACCTCACCCTCC
Sox 9	GAGCAGACGCACATCTC	CCTGGGATTGCCCCGA
Gapdh	TGCACCACCAACTGCTTAGC	GGCATGGACTGTGGTCATGAG

### Glycosaminoglycan quantification

The dimethylmethylene blue (DMMB) assay was used to study the glycosaminoglycans (GAGs) content as previously described.^84^ Briefly, 50 μl of papain‐digested sample harvested at day 21 were added in triplicate to a 96‐well plate and combined with 200 μl of DMMB dye, and the absorbance at 540 nm was immediately read. To determine the GAGs content of the samples chondroitin sulfate from shark cartilage (Sigma) was used as standard.

### Type II collagen quantification

Type II collagen content produced in the scaffolds was quantified by ELISA (Type II Collagen Detection kit #6018; Chondrex, Redmond, WA) according to manufacturer's instruction. Briefly, samples were digested using pepsin in 0.5 M acetic acid: collagen ratio of 1:10 (w/w) for 2 days. Once digested, samples were incubated at 4°C overnight in elastase: collagen ratio of 1:10 (w/w). Then, standard and samples were placed in a precoated 96‐well plate with capture antibodies and incubated for 30 min. The detection antibody was added and incubated for 1.5 h and then washed. The plate was incubated with streptavidin peroxidase for 1 h, washed, and incubated with ortho‐phenyldiamine (OPD) solution for 30 min. A solution of 2 N sulfuric acid was added to stop the reaction, and the content of type II collagen was quantified by absorbance at 490 nm.

### *In vivo* assays

*In vivo* assays were carried out in accordance with the approved guidelines of University of Granada following institutional and international standards for animal welfare and experimental procedure. The Research Ethics Committee of the University of Granada approved all experimental protocols. Experiments were performed in immunocompetent CD‐1 mice and immunodeficient NOD SCID (NOD.CB17‐Prkdcscid/NcrCrl) (NSG) mice purchased from Charles River (Barcelona, Spain). To evaluate the biocompatibility, PCL and b‐TPUe cell‐free scaffolds were transplanted into two independent small subcutaneous pockets made on the back of CD‐1 mice anesthetized by isoflurane inhalation (*n* = 5 per group). In addition, MSCs cell‐laden scaffolds cultured for 21 days were implanted into two independent small subcutaneous pockets created on the back of NSG mice anesthetized by isoflurane inhalation to evaluate engraftment. Cell‐laden or cell‐free scaffolds were implanted in each pocket with a single biomaterial per mouse (b‐TPUe or PCL) (*n* = 5 per group). Animals were maintained in a microventilated cage system with a 12h light/dark cycle with food and water ad libitum. Mice were manipulated within a laminar airflow hood to maintain pathogen‐free conditions. Three weeks later, mice were sacrificed via an overdose injection of anesthetic, and the scaffolds were photographed to evaluate the implantation within the surrounding mouse tissue and recovered for histological analyses. For the histological analysis, samples were dehydrated, embedded in Technovit 7200 and polymerized. The blocks were sectioned with a diamond‐coated band saw (Exakt 310 CP) and then, grounded, and polished with a high precision grinder (Exakt 400). The total histological processing, including Toluidine Blue and Masson staining, were performed by Histology Unit of BIONAND (Málaga, Spain) following the Donath and Bruener cutting/grinding technique.^85^

### Statistical analysis

Statistical calculations were performed using SPSS 13.0 software for Windows (SPSS, Chicago, IL, USA). All graphed data represent the mean ± SD from at least three replicas. Differences between treatments were tested using the two‐tailed Student's *t* test. Assumptions of Student's *t* test (homoscedasticity and normality) were tested and assured by using transformed data sets [log(dependent variable value +1)] when necessary. *p*‐Values <0.05 (*) and *p*‐values <0.01 (**) were considered statistically significant in all cases.

## CONFLICT OF INTEREST

The authors declare no conflicts of interest.

### PEER REVIEW

The peer review history for this article is available at https://publons.com/publon/10.1002/btm2.10192.

## Supporting information


**Data S1** Supporting Information.
