# Supplementary material for: Validation of the 1,4‐butanediol thermoplastic polyurethane as a novel material for 3D bioprinting applications
Source: Bioeng Transl Med. 2020 Nov 14;6(1):e10192. doi: 10.1002/btm2.10192 (PMC7823129; doi:10.1002/btm2.10192)
Supplement: Supplementary file 1 — Data S1 Supporting Information. [file BTM2-6-e10192-s001.docx]

Validation of the 1,4-Butanediol thermoplastic polyurethane (b-TPUe) as a novel material for 3D bioprinting applications

Carlos Chocarro-Wrona^1,2,3,4^, Juan de Vicente^4 5^, Cristina Antich^1,2,3,4^, Gema Jiménez^1,2,4^, Daniel Martínez-Moreno^1,2,3,4^, Esmeralda Carrillo^1,2,3,4^, Elvira Montañez^6,7^, Patricia Gálvez-Martín^8,9^, Macarena Perán^1,2,4,10^, Elena López-Ruiz^1,2,4,10*^, Juan A. Marchal^1,2,3,4 *^.

1. Biosanitary Research Institute of Granada (ibs.GRANADA), University Hospitals of Granada-University of Granada, Granada, Spain.
2. Biopathology and Regenerative Medicine Institute (IBIMER), Centre for Biomedical Research (CIBM), University of Granada, Granada, Spain.
3. Department of Human Anatomy and Embryology, Faculty of Medicine, University of Granada, Granada, Spain.
4. Excellence Research Unit "Modeling Nature" (MNat), University of Granada, Granada, Spain.
5. Department of Applied Physics, Faculty of Sciences, University of Granada, Granada, Spain.
6. Biomedical Research Institute of Málaga (IBIMA), Málaga.
7. Department of Orthopedic Surgery and Traumatology, Virgen de la Victoria University Hospital, 29010 Málaga, Spain.
8. Department of Pharmacy and Pharmaceutical Technology, School of Pharmacy, University of Granada, Granada, Spain.
9. Advanced Therapies Area, Bioibérica S.A.U., Barcelona, Spain.
10. Department of Health Sciences, University of Jaén, Jaén, Spain.

* Corresponding authors.

Contact information:

Corresponding Authors

* Prof. Juan Antonio Marchal, MD, PhD: e-mail: [jmarchal@ugr.es](mailto:jmarchal@ugr.es).

* Elena López-Ruiz; e-mail: [elop@ugr.es](mailto:elop@ugr.es)


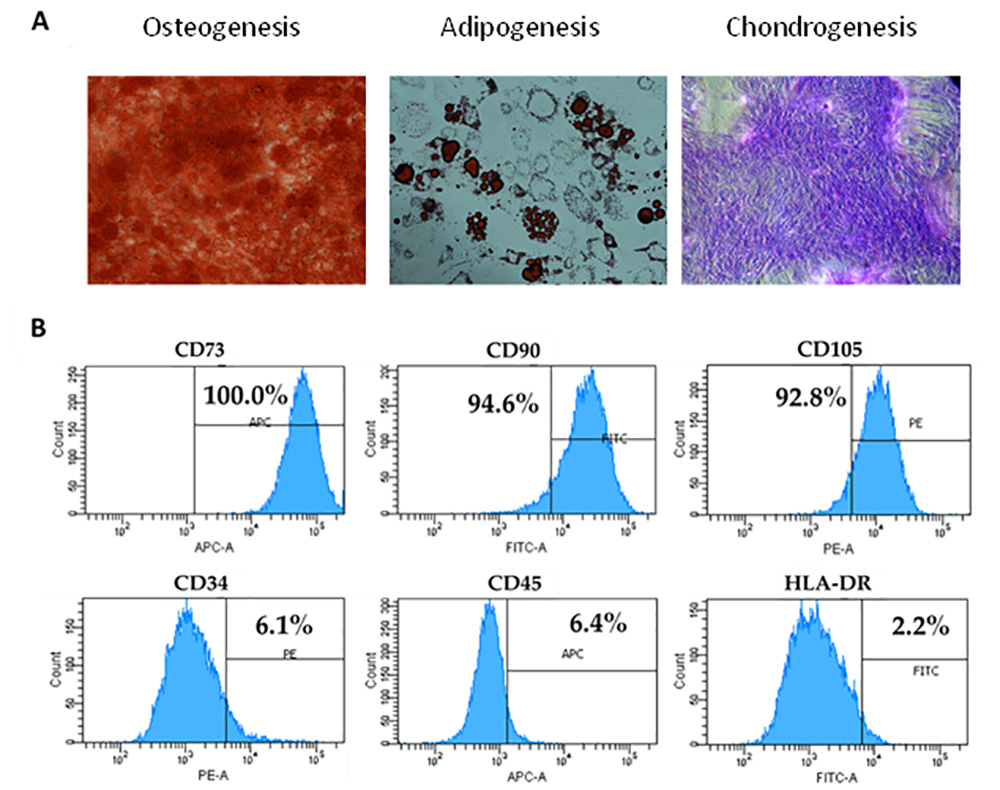


**Supplementary figure 1.** Phenotypic characterization and differentiation potential of MSCs. (A) The differentiation potential of MSCs obtained from lipoaspirate towards osteogenic, adipogenic and chondrogenic lineage was confirmed by alizarin red S, oil red O, and toluidine blue staining, respectively. Scale bar: 100 μm. (B) FACS characterization of MSCs showed a positive expression of surface markers CD73, CD90 and CD105 and negative for CD34, CD45 and HLA-DR
